# Subtractive Immunization as a Method to Develop Respiratory Syncytial Virus (RSV)—Specific Monoclonal Antibodies

**DOI:** 10.3390/antib12040062

**Published:** 2023-09-26

**Authors:** Lotte Jacobs, Kim Stobbelaar, Annick Heykers, Paul Cos, Peter Delputte

**Affiliations:** 1 Laboratory for Microbiology, Parasitology and Hygiene, Infla-Med Centre of Excellence, University of Antwerp (UA), Universiteitsplein 1 S.7, 2610 Antwerp, Belgium; lotte.jacobs@uantwerpen.be (L.J.); kim.stobbelaar@uantwerpen.be (K.S.); annick.heykers@hotmail.be (A.H.); paul.cos@uantwerpen.be (P.C.); 2 Pediatrics Department, Antwerp University Hospital (UZA), Wilrijkstraat 10, 2650 Edegem, Belgium

**Keywords:** RSV, subtractive immunization, monoclonal antibody

## Abstract

Respiratory Syncytial Virus (RSV) is a significant cause of lower respiratory tract infections in the young, the elderly, and in immunodeficient patients. As such, the virus represents an important cause of morbidity and mortality worldwide. Development of monoclonal antibodies against RSV has resulted in a commercial prophylaxis, palivizumab (Synagis^®^), and different antibodies that have improved our understanding of the structure of the viral proteins. In this study, a different immunization technique, subtractive immunization, was evaluated for its applicability to develop RSV-specific antibodies. One hybridoma which produced antibodies with the strongest staining of RSV infected cells, ATAC-0025, was selected for further characterization. This antibody belongs to the IgG1 class, has neutralizing capacity and recognizes the envelope F-protein. The antibody has a broad reactivity against a range of RSV reference strains and clinical isolates.

## 1. Introduction

Respiratory Syncytial Virus (RSV), recently reclassified to the human Orthopneumovirus species, is worldwide one of the leading causes of medically significant viral respiratory tract infections [1,2]. In older children and healthy adults, infections remain mostly asymptomatic or confined to the upper respiratory tract, but in very young children, immunocompromised patients and elderly the infection can evolve to a lower respiratory tract infection, such as acute bronchiolitis or pneumonia, resulting in increased morbidity and mortality [3]. Despite this high medical and societal burden, therapeutic options remain limited to supportive care. However, after more than 60 years and many failed attempts to develop a safe and effective vaccine for this disease, the first FDA-approved RSV vaccines for elderly and pregnant women will soon be marketed [4]. There is also a monoclonal antibody (mAb), palivizumab (Synagis^®^), available for passive immunoprophylaxis, but its use is limited to high-risk populations because of limited efficacy, high cost and the need for multiple monthly injections [5]. Therefore, the long-acting antibody nirsevimab (Beyfortus^TM^) will hopefully be available in a single-shot regimen for all infants aged 0–12 months old as of next winter season [4].

RSV is an enveloped virus with single-stranded non-segmented negative-sense RNA genome belonging to the Pneumoviridae family and can be divided in two antigenic subgroups, namely RSV-A and RSV-B [2]. Strains of these subgroups often co-circulate with alternating predominance of one subgroup [6]. It has a 15.2 kb genome, consisting of 10 genes and encoding for 11 proteins, of which the three surface glycoproteins, the attachment (G) protein, the fusion (F) protein, and the small hydrophobic (SH) protein [2], are the most important. The G protein ensures attachment of the virion to the host cells [7], whereas the F protein mediates fusion of viral and cellular membranes, as well as viral entry, spread and replication [7,8]. The SH protein improves cellular permeability through pore formation [7]. The G and F proteins are the only two RSV antigens to which neutralizing antibody responses are induced [9]. 

mAbs are molecules that bind with high specificity and selectivity to their target and are essential tools in research, but also for therapy and diagnostics. However, it can be challenging to obtain mAbs with desired specificity. In 1975, Köhler and Milstein succeeded in fusing antibody-producing mouse spleen cells with mouse myeloma cells to produce hybridoma cells, which lead to their implementation in various fields, ranging from in vitro immunoassay to immunotherapeutic techniques [10,11,12,13,14]. Although the hybridoma technique can easily be applied to antigens that are produced in a recombinant way, this does not always result in antibodies with the required characteristics [15]. How an antigen is presented during immunization can have a determinative role in the resulting specificity of the antibodies [16]. In RSV research, different immunization strategies have been used in the past, ranging from purified virus and recombinant proteins to vector-based expression of an RSV protein [17,18,19,20]. Especially when infected cells or virus derived from infected cells are used, this usually results in development of an abundance of antibodies against cellular and other immunodominant proteins, making identification of the intended antibodies difficult. Drug-induced subtractive immunization, a less known technique, prevents the occurrence of these common cell-specific mAbs and allows for the development of certain antibodies that are otherwise difficult to obtain, such as mAb against antigens expressed in a cellular context, antigens that need to be expressed in a cellular context for correct conformation or those that are difficult to purify [21]. In this study, we evaluated the application of subtractive immunization to develop antibodies against RSV proteins that are expressed in infected cells and are, therefore, in their native state.

To this end, mice were first made immunotolerant for the human cell line HEp-2, generally used to infect with RSV, in the tolerization phase, which allows a subsequent immunization with RSV-infected HEp-2 cells, expressing all RSV-proteins. Upon hybridoma generation and preliminary characterization, one RSV positive hybridoma, which was named ATAC-0025, was selected and subsequently further characterized. The mAb class, Ab specificity, and affinity for different F-conformations, neutralizing capacity, binding to pre- and postfusion RSV F, and reactivity on Western blot were determined. 

## 2. Materials and Methods

### 2.1. Cells and Viruses

The human epidermoid carcinoma laryngeal cell line, HEp-2 cells, and the African green monkey kidney cell line, Vero cells, were purchased from the ATCC (Manassas, VA, USA) and cultured at 37 °C and 5% CO_2_ in Dulbecco’s modified Eagle medium (DMEM) containing 10% heat-inactivated fetal calf serum (iFCS) and 2% penicillin-streptomycin (Thermo Fisher Scientific, Waltham, MA USA). The T7 RNA polymerase expressing cell line, BSR T7/5 cells, were kindly gifted by K.K Conzelmann (Max-von-Pettenhofer-Institut, Munich, Germany) and were cultivated in Glasgow’s minimal essential medium (GMEM), supplemented with 10% iFCS, 2% minimal essential amino acids and 2% penicillin-streptomycin (Thermo Fisher Scientific).

The RSV clinical isolates A1998/3-2, A2000/3-4, A2001/3-12 were obtained from BEI resources (Manassas, VA, USA) and the BE-ANT-A12/17, BE-ANT-B2/2017 and BE-ANT-B20/2017 clinical strains were isolated from pediatric patients suffering from an RSV bronchiolitis at the Antwerp university hospital by our own group [6]. Both clinical isolates, as well as the RSV reference strain RSV-A2 (BEI resources) were propagated in the HEp-2 cell line. The RSV reference strain RSV-B1 (BEI resources) was cultivated using the Vero cell line. For the preparation of the RSV strains, an 80% confluent monolayer of either HEp-2 cells or Vero-cells was inoculated with a suspension of the respective RSV strain in DMEM without iFCS for 2 h. Afterwards the cells were maintained at 37 °C and 5% CO_2_ in DMEM containing 2% iFCS. Virus collection was performed as soon as a clear cytopathic effect was visible throughout the cell culture, after which cells are scraped and pelleted at 1000 g and viral supernatants are aliquoted, snap frozen, and stored at −80 °C until further use.

### 2.2. HEp-2 Immunotolerance

To avoid an antibody response of the mice to HEp-2 cellular antigens, 6-week-old female BALB/c mice (Janvier Laboratories, Le Genest-Saint-Isle, France) were made tolerant for HEp-2 cells, as previously described by Matthews et al. [22]. Briefly, the mice were injected intraperitoneally (i.p.) with a suspension of 10^7^ cells in 300 µL sterile phosphate-buffered saline (PBS). At 10 min, 24 h, and 48 h after the injection of the cells, the mice were i.p. treated with a suspension of 100 mg/kg cyclophosphamide (Sigma, Burlington, MA, USA) in a total volume of 500 µL PBS. Serum samples were taken right before and 1 week after treatment and tested on reactivity towards HEp-2 cells by means of immunofluorescent staining of non-infected HEp-2 cells. The entire treatment was repeated every 3 weeks, until no reactivity towards HEp-2 cells was observed. 

The animal experiments were evaluated and authorized by the Ethical Committee for animal testing of the University of Antwerp (Permit number: 2015-28). All mouse experiments were carried out following the appropriate regulations. Food (Carfil, Oud-Turnhout, Belgium) and drinking water were provided ad libitum.

### 2.3. RSV-Immunization

After tolerization, the HEp-2 tolerant mice were i.p. injected with 10^7^ HEp-2 cells infected with RSV at an average infection rate of 75%, dissolved in 150 µL complete Freund’s adjuvants. A booster was administered after two weeks. Two weeks later, sera were collected and tested for an RSV-specific antibody response and the absence of reactivity with HEp-2 cells. Once an antibody response against RSV was observed, the mice were i.p. injected with 10^7^ infected HEp-2 cells dissolved in 150 µL PBS a final time. Euthanasia was performed 6 days later, and the spleen was collected.

### 2.4. Production and Screening of Hybridomas

The spleen cells (10^8^ cells) were fused with SP2/0 (10^7^ cells) using PEG 1500, as previously described [12]. Fused cells were selected in hypoxanthine-aminopterin-thymidine (HAT)-Iscove’s Modified Dulbecco’s Medium (IMDM). After an incubation period of 2 weeks, growing colonies were selected for secreting anti-RSV antibodies. RSV-specific mAbs in the hybridoma supernatant were demonstrated by immunofluorescent staining. At 75% confluency, the supernatant was collected and was added to RSV-infected and -uninfected HEp-2 cells, after fixation with 4% paraformaldehyde (PF) and permeabilization with 0.5% Triton x-100. After incubation with undiluted supernatants for 1 h at 37 °C, cells were washed three times with PBS. Subsequently, a 1/1000 diluted donkey anti-mouse AF488 labeled antibody (Life Technologies, Carlsbad, CA, USA) in PBS was added for 1 h at 37 °C. After washing the cells with PBS and a short incubation with 4′,6-diamidino-2-phenylindole (DAPI) for nucleus staining, the cells were evaluated under the fluorescence microscope. The selected hybridomas were cloned by limiting dilution.

### 2.5. Antibody Purification and Determination of mAb Class

The selected ATAC-0025 mAb was purified from concentrated supernatants using the NAb spin kit (Thermo Fisher Scientific), according to the manufacturer’s instructions. The resulting purified antibody was subsequently concentrated by means of Pierce Protein Concentrators, PES (Thermo Fisher Scientific), to obtain a concentration 0.5 mg/mL. The mAb class was determined with a commercial mouse isotyping kit MMT1 (Bio-Rad, Hercules, CA, USA). The different Ig isotype classes and light chains were determined through immune-chromatography.

### 2.6. Determining the Specificity of ATAC-0025 

HEp-2 cells were seeded in 96-well plates at a concentration of 1.75 × 10^5^ cells/mL 24 h prior to infection. Next, the HEp-2 cells were infected with the RSV A2 reference strain at an MOI of 0.05 and incubated in DMEM with 2% iFBS at 37 °C and 5% CO_2,_ after a short incubation in DMEM without FBS. After 48 h, infected and uninfected cells were fixed with 4% PF, and stained with purified ATAC-0025. In order to evaluate surface expression of the epitope of ATAC-0025, only half of the cells were permeabilized with 0.5% Triton X-100 prior to addition of the mAb. Palivizumab and a mouse IgG1 antibody were used as control, to exclude aspecific binding. Cells were subsequently stained with secondary AF488-labeled goat-anti-human and goat-anti-mouse IgG antibodies, as appropriate.

Once surface expression of the ATAC-0025 binding epitope was demonstrated, preseeded HEp-2 cells were transfected with plasmids expressing, respectively, the RSV-A2 F or G protein using FuGENE HD Transfection Reagent (Promega, Madison, WI, USA) according to the manufacturers’ instructions. Immunofluorescent staining was performed after 48 h, as described above. Images were obtained using the TECAN Spark Cyto 600 live-cell imaging system (Mechelen, Belgium) at 10× magnification.

### 2.7. Cross-Reactivity to Different RSV Strains

Pre-seeded 96-well plates containing monolayers of HEp-2 cells at a concentration of 1.75 × 10^5^ cells/mL were inoculated with 7 different clinical RSV isolates, A1998/3-2, A2000/3-4, A2001/3-12, BE-ANT-A1/17, BE-ANT-A12/17, BE-ANT-B2/2017, and BE-ANT-B20/2017, as well as the reference strains RSV A2, A2Line19F and B1 at an MOI of 1, for 2 h at 37 °C and 5% CO_2_ in DMEM. Afterwards, DMEM containing 4% FBS was added to the inoculum in a 1:1 ratio and the cells were incubated for another 24 h at 37 °C and 5% CO_2_. The cells were subsequently fixed, permeabilized, and immunofluorescently stained as described above. Images were obtained using the TECAN Spark Cyto 600 live-cell imaging system (Mechelen, Belgium) at 10× magnification.

### 2.8. ELISA

Pierce^TM^ Nickel Coated Plates (Thermo Fisher Scientific) were coated with pre- and postfusion stabilized F proteins (kindly gifted by J. McLellan [23,24]) at a concentration of 1 µg/mL for 1 h at room temperature on a shaking plate. Coated plates were blocked with 1% bovine serum albumin (BSA) overnight and washed four times with PBS-Tween (PBS-T). 1:3 serial dilutions of ATAC-0025, palivizumab (pre- and postfusion F-specific mAb) and D25 (prefusion F-specific mAb) were incubated for 1 h at room temperature. Analyses were performed in triplicate. Next, plates were washed four times with PBS-T and incubated with goat anti-human HRP for palivizumab and D25 or goat anti-mouse HRP for ATAC-0025 (ThermoFisher Scientific) for 1 h at room temperature. After four final washes with PBS-T, 3,3′,5,5′-tetramethylbenzidine (TMB) (Sigma) was added to the plates and incubated at room temperature for 30 min. The colorimetric reaction was stopped with a stop solution (2 N sulfuric acid). Absorbance was measured at 450 nm using a spectrophotometer (GloMax Discover, Promega, Madison, WI, USA).

### 2.9. Western Blot 

RSV A2 virus pellets were acquired by ultracentrifugation (90 min, 20,000 rpm, 4 °C) (Optima™ XPN) of RSV A2 infected HEp-2 cells. Pellets were resuspended in Hanks’ Balanced Salt Solution (HBSS). Western blot samples were prepared by mixing viral suspension with Laemmli buffer (1:1), with or without addition of β-mercapthoethanol (for reduction). Samples were denatured by boiling the mixtures for 5 min, and were loaded and separated on 4–20% polyacrylamide gels (Bio-Rad). Proteins were transferred to an Immobilon-P transfer membrane (Millipore, Burlington, MA, USA). The RSV proteins were incubated with concentrated hybridoma supernatant, and subsequently with HRP-conjugated goat anti-mouse antibody (Thermo Fisher Scientific). As a control reaction, the RSV F proteins were stained with palivizumab and HRP-conjugated goat anti-human IgG (Thermo Fisher Scientific). Protein bands were visualized with Pierce^TM^ ECL Western blotting substrate (Thermo Fisher Scientific).

### 2.10. Neutralization Capacity

To determine the neutralizing capacity, 1:2 dilution series of ATAC-0025 and palivizumab were made starting at a concentration of 50 µg/mL. These dilution series were made in a suspension of DMEM without iFCS containing 1500 PFU/mL of RSV. The resulting suspensions were incubated for 1 h at 37 °C and 5% CO_2_. The Ab-virus solutions were then added to 80% confluent monolayers of HEp-2 cells and, after an incubation of 2 h at 37 °C in 5% CO_2_, the cells were washed with PBS. The HEp-2 cells were subsequently incubated at 37 °C and 5% CO_2_ for 3 days, with an overlay consisting of 0.6% avicel (FMC Biopolymer, Philadelphia, PA, USA) in DMEM containing 10% iFCS. Afterwards, the avicel-containing medium was removed, cells were washed twice, and subsequently fixation was undertaken for 20 min with 4% PF, followed by permeabilization with 0.5% triton X-100. For visualization of plaques, an immunostaining with palivizumab as primary antibody was performed, followed by the addition of a secondary HRP conjugated goat anti-human antibody (Life Technologies) for 1 h and, lastly, 1-step chloronapthol substrate solution (Thermo Fisher Scientific), which generates a blue color upon conversion.

## 3. Results

### 3.1. Mouse Immunization and Hybridoma Production 

Six BALB/c mice were made immunotolerant to HEp-2 cells by repeated injection of non-infected HEp-2 cells, followed by cyclophosphamide treatment. Mice sera were tested for reactivity with HEp-2 cells, and only mice showing no reactivity were selected for subsequent immunization with RSV-infected HEp-2 cells. One week after the second booster immunization, only one mouse with anti-RSV antibody responses received a final booster injection. Six days later, this mouse was sacrificed, and the spleen was collected for the production of hybridomas.

When the hybridomas were sufficiently grown, supernatant was collected and tested with an immunofluorescence staining against RSV-infected HEp-2 cells. Cloning of the selected hybridoma populations by limiting dilution resulted in stable hybridomas that produced mAbs. One hybridoma with the strongest staining of RSV-infected cells was selected. The antibody produced by this hybridoma was named ATAC-0025, and was further characterized. Using a commercial isotyping kit based on immune-chromatography, ATAC-0025 was isotyped as IgG1.

### 3.2. Specificity of ATAC-0025 Antibody

**Figure 1:**
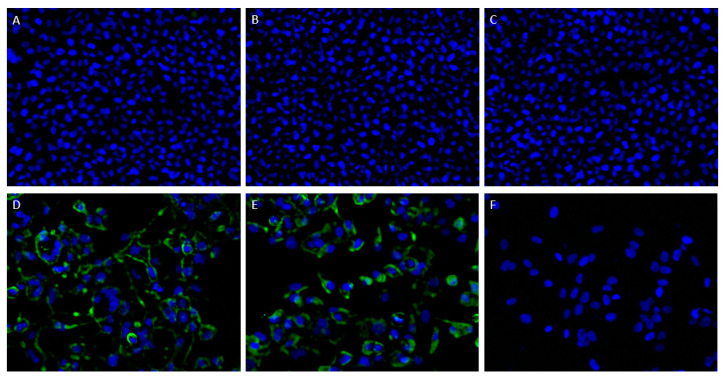
Immunofluorescent staining of non-infected (**A**–**C**) and RSV-infected (**D**–**F**) HEp-2 cells confirms the specificity of ATAC-0025 for an RSV protein. Nuclei were stained with DAPI (blue), surface protein was stained with ATAC-0025 (**A**,**D**), palivizumab (**B**,**E**) and a mouse IgG1 control antibody (**C**,**F**).

As ATAC-0025 showed a strong staining pattern in RSV-infected HEp-2 cells, further characterization of this mAb was undertaken. This staining pattern was absent in the uninfected HEp-2 cells, confirming its specificity for an RSV protein. Palivizumab and a mouse IgG1 antibody were included as controls (Figure 1). No staining was seen with the mouse IgG1 antibody, thereby excluding aspecific binding of ATAC-0025.

**Figure 2:**
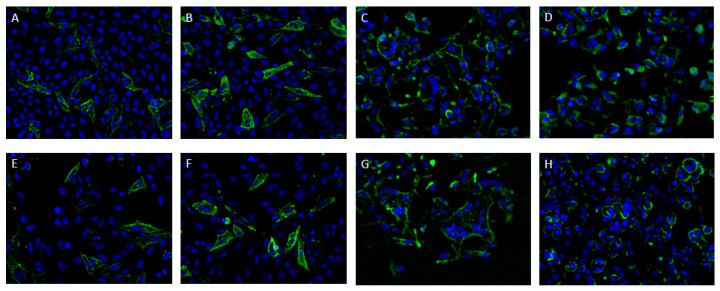
Immunofluorescent staining of RSV-infected (**A**,**B**,**E**,**F**) and F protein transfected (**C**,**D**,**G**,**H**) HEp-2 cells. (**A**) After permeabilization, a clear staining pattern is seen with ATAC-0025. (**E**) Without permeabilization, surface staining is still present, indicating that ATAC-0025 recognizes an RSV envelope protein that is also expressed on the surface of infected cells. (**B**,**F**) Staining with palivizumab as a reference, with (**B**) and without (**F**) preceding permeabilization. (**C**,**D**,**G**,**H**) HEp-2 cells that were transfected with the RSV-F show a clear staining pattern with ATAC-0025 (**C**,**G**), comparable to staining with palivizumab (**D**,**H**). Nuclei were stained with DAPI (blue).

When the infected HEp-2 cells were not permeabilized prior to staining, the staining still resulted in a clear surface staining, which indicates that the antibody recognizes an RSV envelope protein. To identify this protein, the hybridoma supernatant was tested on transfected HEp-2 cells, expressing either the F or G protein. Staining with polyclonal goat anti-RSV antibodies confirmed a successful transfection and expression of the recombinant proteins. ATAC-0025 only stained the cells expressing the RSV F-protein (Figure 2). 

### 3.3. Cross Reactivity 

**Figure 3:**
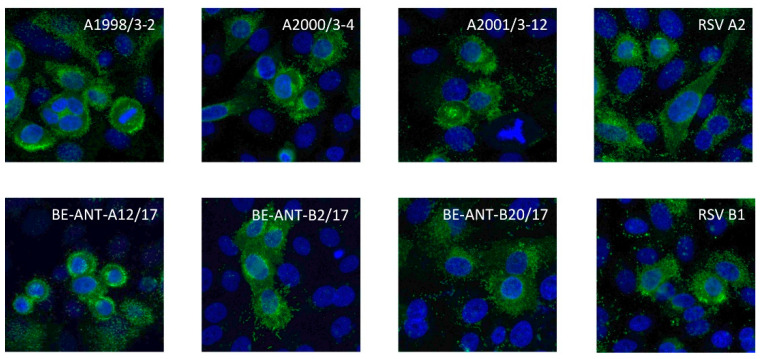
Immunofluorescent staining of HEp-2 cells infected with different RSV strains with ATAC-0025. Nuclear staining was performed with DAPI.

To further evaluate the reactivity of ATAC-0025 with the F protein from different RSV strains, immunostaining of HEp-2 cells infected with different clinical RSV isolates and RSV reference strains was performed. All viruses, both RSV-A and RSV-B strains, were successfully stained with ATAC-0025, suggesting that the mAb reacts with a conserved epitope in both older reference isolates and more recent clinical isolates (Figure 3).

### 3.4. Pre- and Postfusion-Specific ELISA

**Figure 4:**
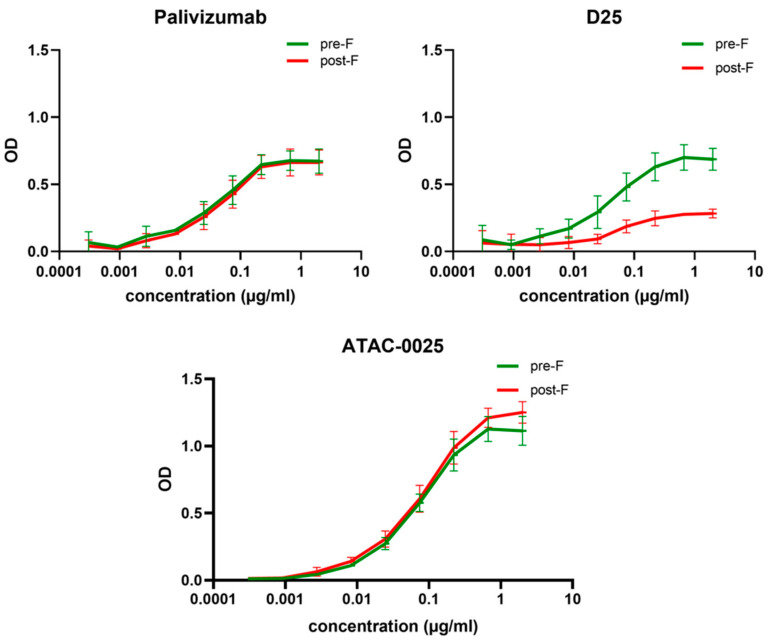
Pre- and postfusion-specific ELISA. ATAC-0025 pre- and/or postfusion conformation affinity was determined in ELISA. Plates were coated with pre- and post-F stabilized proteins. Palivizumab and D25 served as control antibodies for pre/post F and pre-F specific conformations, respectively. The experiment was performed in triplicate, and values are presented as means with SD (n = 3).

To evaluate specificity of ATAC-0025 for the pre- and/or postfusion RSV F protein, plates were coated with stabilized pre- and postfusion F proteins ([23,24]). Palivizumab (pre- and postfusion F specific mAb) and D25 (prefusion F specific mAb) were used as references with known binding specificities. While D25 clearly has a binding preference for the prefusion F, ATAC-0025, just like palivizumab, equally binds to pre- and post-F proteins (Figure 4).

### 3.5. Reactivity of mAb ATAC-0025 in Western Blot

**Figure 5:**
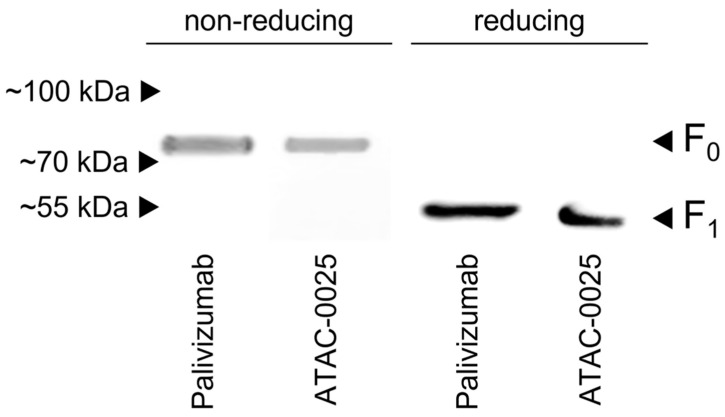
Detection of the RSV F protein on Western blot. Purified RSV proteins were separated under non-reducing and reducing conditions, transferred to a PVDF membrane, and stained with ATAC-0025 antibody and palivizumab as a reference.

The reactivity of the antibody in a Western blot assay was determined (Figure 5). The supernatant from RSV A2 infected HEp-2 cells was pelleted through ultracentrifugation, lysed in reducing and non-reducing conditions, loaded on the gels for separation and transferred to the blotting membrane. In non-reducing conditions, a clear band was observed at the site of the F_0_, while in reducing conditions, reactivity was observed at the expected size of the F_1_ subunit.

### 3.6. Neutralization Assay 

Since the mAb is specific for the RSV F envelope protein, which is a target for neutralizing antibodies [7], the neutralizing capacity for the reference strains RSV-A2, RSV-A2L19, and RSV-B1 and a selection of previously characterized RSV isolates was tested, and compared to the neutralizing capacity of palivizumab, which is currently used as prophylaxis in high-risk infants. The concentration at which the antibodies successfully neutralized 50% of the virus was calculated for each RSV strain. The ATAC-0025 antibody can neutralize all RSV strains tested, although the neutralization capacity is low and a clearly higher antibody concentration (>10 fold) is needed to achieve 50% neutralization compared to palivizumab (Table 1). 

## 4. Discussion

In the present study, it was demonstrated that drug-induced subtractive immunization is an efficient and cost-effective tool for generating mAb against RSV proteins expressed in infected cells, which can be expected to be closer to their native conformation compared to purified recombinant proteins. To this end, RSV-specific hybridomas were produced by immunizing mice with RSV-infected HEp-2 cells, which is in contrast to previous studies where immunization happened with purified virus, isolated proteins or peptides, or vector-based expression of an RSV protein [17,18,19,20]. The methodology used here allows for the generation of antibodies toward the native state of the F protein expressed on the cell surface and, therefore, avoids tedious and time-consuming steps for isolation, purification, and recombinant engineering of proteins. Furthermore, the native confirmation of these viral proteins is preferred, as this represents natural infection and may therefore elicit mAb that would otherwise could not be developed.

Subtractive immunization works by first inducing tolerance for a specific set of antigens, the tolerogens, toward which there is no desire in obtaining mAbs. Next, it allows antibody production against novel antigens that are present in the background of tolerogens, the immunogens. Immunogens generally carry rare and/or poorly expressed epitopes. Here, tolerance was induced for cellular antigens by repeatedly injecting HEp-2 cells combined with cyclophosphamide treatments. Cyclophosphamide is an immunosuppressive drug with anti-proliferative activity. Thus, stimulated B- and T- lymphocytes that proliferate after the administration of the tolerogen are thereby specifically eliminated [25,26,27,28]. In this study, tolerance was induced against HEp-2 cells, after which mice were immunized with RSV-infected HEp-2 cells. Mouse serum was collected after each tolerization cycle with cyclophosphamide. Only when no reaction against HEp-2 cells was monitored, the mice were selected for subsequent immunization with RSV-infected HEp-2 cells and hybridoma development. Of the RSV positive hybridomas that were generated, one hybridoma was selected as it demonstrated intense staining of RSV-infected cells, and was further characterized.

The ATAC-0025 mAb had a clear specificity for an RSV envelope protein, which was demonstrated by the absence of immunofluorescent signal in non-infected cells and the presence of a clear staining pattern, even without permeabilization. After transfection of HEp-2 cells with the major envelope proteins F and G, and subsequent immunostaining, only cells expressing F were stained, indicating the F protein, a major target for vaccine and mAb development, as antigenic target for ATAC-0025. Further characterization shows the capacity of ATAC-0025 to recognize a broad variety of RSV reference strains and recent clinical isolates, generating possibilities for its use in diagnostic and therapeutic settings. Since ATAC-0025 antibody recognizes F, which exists in a pre- and postfusion conformation, binding with both pre- and post-F was tested in a specific ELISA. The mAb demonstrates a slight but non-significant preference for the post-F conformation and detects RSV F in Western blot in both non-reducing and reducing conditions, suggesting that it recognizes a conformational epitope.

The advantages of the methodology described here have already been reported elaborately, and mainly entail the possibility to reliably and controllably generate mAb against complicated or unclear antigens, antigens that are difficult to be extracted or purified, antigens that are weakly immunogenic, or those that need to be expressed in a cellular context for correct conformation [25]. The immunization technique also avoids setting up sometimes difficult and time-consuming purification protocols to obtain pure recombinant proteins. Finally, the technique is also very useful to make antibodies that can distinguish closely related antigens, such as antibodies used to discriminate between different serotypes, or antibodies that react with epitopes differentially present in two forms of the same protein [26]. Disadvantages of this technique are the higher cost and the possibility of mouse death, especially because of the immunosuppressant drug used in the tolerization phase, i.e., cyclophosphamide in this study [25]. Also, besides killing B-cell clones in proliferation, cyclophosphamide also kills helper T-cells, which are necessary for B-cell maturation and differentiation [25].

Besides drug-induced subtractive immunization, other approaches for subtractive immunization have been described, including neonatal tolerization, high-zone tolerance, and masking subtractive immunization. Each of these techniques has their own pros and cons and only a few reports have compared these techniques with each other [27]. A combination of two or more of these methods might result in better antibodies. In fact, Ensrud and Hamilton combined neonatal tolerization and drug-induced tolerization to generate maturation-specific sperm surface molecules. Combining different subtractive immunization techniques might be beneficial by altering the immune response in various ways, thereby possibly resulting in antibodies with even higher specificities, yet these are more difficult to implement [25].

The limitations of our study are that while ATAC-0025 has been shown to broadly neutralize different RSV isolates, both historical reference strains as well as currently circulating clinical isolates, this neutralization is clearly less efficient compared to palivizumab. One of the hypotheses for this is that the epitope of the ATAC-0025 mAb on the RSV F protein is less involved in neutralization. However, we do not know the exact location of this epitope. To elucidate the specific epitope, additional experiments with palivizumab and other well-characterized mAb against RSV with known epitopes would be needed. Another limitation is the use of cyclophosphamide in the tolerization phase, as this immunosuppressive drug could have negative effects on the mice when not used appropriately [21]. However, cyclophosphamide induced subtractive immunization has been widely used in research. It has been proven to be useful in several scientific areas and applications, including cancer research and identification of food contamination [29,30,31,32].

To the best of our knowledge, this report is the first to describe the use of subtractive immunization in generation of mAb against RSV proteins. Not only did we confirm the high suitability of drug-induced subtractive immunization for the production of mAb against RSV proteins in native conformation, we also showed that the ATAC-0025 mAb produced here was able to broadly recognize the RSV F protein of different RSV reference strains and clinical isolates, in both pre- and postfusion states, thereby creating various possibilities for its use in downstream applications.

## Figures and Tables

**Table 1:** Comparison of the neutralizing capacity of ATAC-0025 with that of palivizumab, for different RSV isolates. Neutralization is expressed as the antibody concentration required to neutralize 50% of the RSV-particles present in 1500 PFU/mL RSV dilution. SD = standard deviation.

	ATAC-0025		Palivizumab	
	Concentration with 50% Neutralization (µg/mL)	SD	Concentration with 50% Neutralization (µg/mL)	SD
A2	6.32	±0.45	0.20	±0.07
B1	1.42	±0.13	0.10	±0.01
A2Line19	2.09	±0.08	0.06	±0.01
A1998/3-2	3.91	±0.40	0.12	±0.00
A2000/3-4	5.53	±0.38	0.29	±0.00
A2001/3-12	1.13	±0.04	0.11	±0.02

## Data Availability

The dataset supporting the conclusions of this article is included within the article.
